# Ultrasound-Promoted Abatement of Formaldehyde in Liquid Phase with Electrospun Nanostructured Membranes: The Synergy of Combined AOPs

**DOI:** 10.3390/nano13030435

**Published:** 2023-01-20

**Authors:** Carlo Boaretti, Martina Roso, Michele Modesti, Alessandra Lorenzetti

**Affiliations:** Department of Industrial Engineering, University of Padova, Via Marzolo 9, 35131 Padova, Italy

**Keywords:** formaldehyde, titanium dioxide, photocatalysis, ultrasound, Fenton, AOPs, synergy, nanofibers

## Abstract

The present work investigates the effect of ultrasounds in the performance of combined advanced oxidation processes (AOPs) on the degradation of formaldehyde (HCHO)-polluted aqueous solutions for potential application in wastewater treatment. Different heterogeneous nanostructured catalysts based on TiO_2_ and FeSO_4_ for photocatalysis and the Fenton process were employed after electrospray deposition on electrospun nanofibrous membranes. Such systems were tested, without the use of any added hydrogen peroxide, by varying the combinations among the selected AOPs in a batch reactor configuration. The results show that, in the absence of a Fenton reaction, ultrasounds provided a significantly increased formaldehyde photocatalytic abatement, probably by increasing the concentration of active species through a different set of reactions while providing a favorable mass transfer regime by the cavitational effect. Due to the faster kinetics of the photo–Fenton process, thanks to its partial homogeneous nature, such a beneficial effect is more limited for the sono–photo–Fenton configuration. On the other hand, the employment of a sono–photocatalytic–Fenton process revealed a synergic effect that provided the best results, reducing the formaldehyde concentration to less than 99% after 240 min. Further analysis showed that, due to a mutual influence, only a tailored TiO_2_/FeSO_4_ ratio on the membranes was able to display the best performance.

## 1. Introduction

Formaldehyde (HCHO) is the simplest chemical compound of the aldehyde family, but at the same time, it is one of the most produced chemicals all over the world, with a global market that reached about 22.6 million tons in 2020 [1]. Such high production volumes are justified by its widespread employment as a building block for the synthesis of some of the most important synthetic thermosetting resins (melamine–formaldehyde, urea–formaldehyde, and phenol–formaldehyde) and for the production of important chemicals such as methylene diphenyl diisocyanate for polyurethane production and functional polyalcohols, such as pentaerythritol and 1,4-butanediol. Therefore, its common use in the chemical industry in large quantities, combined with its high toxicity, has led to its classification as a toxic industrial chemical of high hazard [2] due to its potential involvement in terrorist events [3]. Chemical production plants can produce wastewater with up to 10^4^ mg/L of formaldehyde [4], which is difficult to treat, even in the range of 10^2^ mg/L using conventional biological processes due to the adverse effect on the cellular structure of microorganisms [5,6]. Such evidence requires the employment of alternative degradation strategies to induce formaldehyde decomposition [7,8,9,10,11,12] and limit its adverse effects on human health and living organisms.

Advanced oxidation processes (AOPs) is a collective term embracing a wide set of homogeneous and/or heterogeneous abatement techniques that rely on the generation of reactive oxidant species to convert different types of recalcitrant pollutants, such as pharmaceutical, dyes, pesticides, and so on, into a more biodegradable form or up to their complete mineralization [13]. To such end, AOPs involve the use of strong oxidizing agents, a specific catalyst or photocatalyst, and different energy vectors, such as ultraviolet (UV) light irradiation, ultrasound (US), or electrical sources, under relatively mild operating conditions. Despite the great debate around the specific species involved in the degradation mechanism, it is generally considered that the hydroxyl radical plays a pivotal role thanks to its high oxidizing potential and non-selective nature, which is useful for the treatment of different types of organics [14]. Therefore, the proper exploitation of such techniques is based on the maximization of the production of such species, combined with relatively high reaction rates and possible complete or at least partial mineralization of the targeted pollutant into non-toxic compounds. From this point of view, the use of single AOPs can suffer from many possible limitations that still hinder their applicability at an industrial scale [15]. For example, photocatalysis with suspended particles is known to suffer from mass transfer limitations, high electron–hole recombination rates, and possible particle agglomeration [16]; the Fenton process is affected by a slow rate of Fe^3+^ reduction to Fe^2+^, high dependence on the pH, the generation of sludge, and the consumption of expensive hydrogen peroxide (H_2_O_2_) [17,18] while ultrasound can provide very limited pollutant degradation, representing an expensive technology from an economic point of view [19]. However, while an exhaustive solution for a real breakthrough is yet to come, it has been recognized that a possible compromise to solve some of these issues can be found in the proper coupling of these AOPs as a possible way to also find synergic effects [20,21,22,23,24,25].

In such a context, the use of heterogeneous systems based on membranes has been recognized as a promising approach for wastewater treatment [26]. In this sense, electrospun nanofibrous membranes are considered an interesting solution in water filtration due to an ideal combination of different properties in terms of mechanical resistance, high surface area, porosity, and wettability [27]. Despite this, the main application of these types of membranes is still related to filtration, the adsorption of contaminants and heavy metals, and the photocatalytic degradation of organic compounds [28,29,30,31]. A less studied solution is the use of these membranes with a combination of different AOPs. For this purpose, among the different polymeric substrates that can be employed, PAN has been indicated as a viable support for both photocatalysts [32,33] and photo–Fenton [34,35] catalysts thanks to its high UV stability and propensity for surface functionalization.

Many studies dealing with different combinations of AOPs for the degradation of formaldehyde aqueous solution are present in the literature, often focusing on the analysis of the effect of different process parameters. Kajitvichyanukul et al. [36] compared different AOPs based on photocatalysis and Fenton reactions for the abatement of highly concentrated formalin solution. They observed that the photo–Fenton system had the best performance both in terms of formaldehyde abatement and complete mineralization rate. In a second study [37], the same authors investigated the photo–Fenton degradation of formalin in lab-scale experiments with UV light by analyzing the effects of different parameters on the oxidation rates of both formaldehyde and methanol. They observed that formaldehyde removal was less affected by pH, although a pH of 2.6 was necessary to provide the best efficiency, while generally an increase in both the concentrations of Fe^3+^ ions and H_2_O_2_ was able to increase the degradation rate. From their analysis, it was possible to conclude that methanol was found to be more difficult to oxidize when in competition with formaldehyde in the aqueous stream to be treated. Sekiguchi et al. [38] analyzed the degradation of model aldehydes such as formaldehyde and benzaldehyde to assess the possible synergistic effect between different techniques of advanced oxidation, such as ultrasounds, UV photocatalysis, and sono–photocatalysis. The results of the study highlighted that the sono–photocatalytic process was able to display the best performance with a first-order degradation rate and a synergic effect on both of the two analyzed pollutants. Liang et al. [39,40] analyzed the kinetics of formaldehyde degradation using UV–Fenton oxidation, identifying a suitable model for describing the evolution of formaldehyde concentration as a function of the concentrations of Fe^2+^ and H_2_O_2_ in the photochemical reactor. In their conclusions, they highlighted that the main intermediate product of oxidation was formic acid, whose further oxidation was the main rate-limiting step of complete mineralization. Guimaraes et al. [41] compared the degradation efficiency of formaldehyde with the employment of different abatement techniques. They observed that formaldehyde was not possible to degrade by photo-oxidation alone, while peroxidation had a limited effect due to difficulty with the degradation of the formic acid generated as an intermediate oxidation product. In terms of performance, the degradation rates for both UV/H_2_O_2_ and photo–Fenton were similar, while the latter was found to produce a faster decrease in the dissolved organic concentration for highly concentrated formaldehyde solutions. Tymoyrimoghadam et al. [42] degraded formaldehyde in aqueous solutions with the use of a UV/S_2_O_8_^2−^ process in a photoreactor operated in rotary mode. In their experiments, a certain dependence on pH was noted, which favored a higher removal efficiency under acidic conditions, while specific persulfate-to-formaldehyde ratios were identified to obtain the best results. The specific effects of anionic species on the removal efficiency were also discussed. Deniz et al. [43] analyzed formaldehyde degradation using H_2_O_2_ as an oxidant species under UV and fluorescent light irradiation. They noticed a higher dependence of the performance on pH by using fluorescent light, but the best performance was identified for conditions of a neutral pH with a combination of UV light and a suitable mass ratio between formaldehyde and H_2_O_2_. Recently, Lai et al. [44] explored an in situ degradation of formaldehyde by the electro–Fenton process using nitrogen-activated carbon to promote both H_2_O_2_ and formaldehyde adsorption. Through nitrogen doping, they enhanced the degradation of formaldehyde by promoting Fe^2+^ regeneration to improve the production of ^•^OH at the cathode and achieve a higher probability of interaction with the adsorbed HCHO. With this solution, they were able to reach a 49% increase in degradation compared to the unmodified cathode.

The traditional approach in many of these studies has been to improve the degradation rate of formaldehyde removal but without a specific combination of techniques to achieve both a synergic effect and a partial overcoming of the limitations of each single AOP adopted. In this work, we present the use of such a combined strategy, never reported before, to degrade low concentrations of formaldehyde (90 ppm) in aqueous solutions. Starting from the conclusions of our previous work [45], we adopted a membrane substrate to immobilize the catalytic system on a porous support to avoid its suspension, boosting the production of radical species using both the Fenton reaction and UV light without any added H_2_O_2_, along with the introduction of ultrasound irradiation. More specifically, we investigated the effect of the latter as it can provide improvements both in terms of mass transfer conditions and degradation of the selected pollutant. The main aim was to analyze if an additive or synergic effect can arise on both the formaldehyde degradation rate and the final conversion by a specific combination of processes involving the use of ultrasound. The optimal catalytic system composition was also considered, with reference to the best-performing combination of AOPs involved.

## 2. Materials and Methods

### 2.1. Materials

Polyacrylonitrile (PAN, Mw = 150,000 g/mol, Sigma Aldrich, St. Louis, MO, USA) was used as a nanofibrous support for catalyst deposition and electrospinning, using *N*,*N*-dimethylformamide (DMF, Sigma Aldrich, St. Louis, MO, USA) as a solvent. TiO_2_ powder Aeroxide^®^ P90 (d = 14 nm, BET = 90 m^2^/g, 90:10 anatase/rutile ratio, crystal size = 11 nm [46]) was supplied by Evonik (Essen, Germany) and used for the photocatalytic tests, while ferrous sulfate heptahydrate (FeSO_4_·7H_2_O) was supplied by Sigma Aldrich (St. Louis, MO, USA) and employed as a catalyst for all the tests involving the Fenton reaction. The catalysts’ immobilization on the surfaces of PAN nanofibers was realized by electrospraying the corresponding dispersions, using ethanol (Sigma Aldrich, St. Louis, MO, USA) as a solvent and Dynasylan^®^ 4144 (Evonik, Essen, Germany) as a dispersing agent. A 90 ppm formaldehyde aqueous solution was employed for all the abatement tests and realized by the dilution of formalin (37% *w*/*w* of formaldehyde and 10% *w*/*w* of methanol), supplied by VWR Chemicals (Radnor, PA, USA), with MilliQ water. Whenever the Fenton reaction was involved, the pH of such a solution was adjusted to a value of 3 with the use of a 0.2 N sulfuric acid solution (H_2_SO_4_ at 98% *w*/*w*, Sigma Aldrich, St. Louis, MO, USA), as such a process is known to be highly favored under these conditions [47]. For the quantification of the formaldehyde concentration, a specific derivatization method was employed using o-(2,3,4,5,6-pentafluorobenzyl)hydroxylamine hydrochloride (PFBHA-HCl) purchased from Alfa Aesar (Haverhill, MA, USA) and 1,2-dibromopropane at 97% *w*/*w* as internal standards (Sigma Aldrich, St. Louis, MO, USA) and using hexane (Sigma Aldrich, St. Louis, MO, USA) for the extraction process.

### 2.2. Methods

Thermogravimetric analysis (TGA) was the technique employed for the quantification of the amount of photocatalyst deposited on the surface of the electrospun composite membranes. The analysis was carried out with an SDT-Q600 apparatus (TA Instruments, New Castle, DE, USA) equipped with alumina pans, employing a 100 cc/min airflow and a 20 °C/min heating rate from room temperature up to 900 °C. The quantification of the residual formaldehyde concentration was carried out according to the U.S. Environmental Protection Agency (EPA) method 556 [48] using a derivatization procedure with PFBHA-HCl on samples withdrawn from the aqueous solution employed during the degradation tests. The oxime derivative formed was extracted from water with hexane and processed with an acidic wash step before being analyzed by gas chromatography–mass spectroscopy (gas chromatograph TRACE 1300^®^ directly coupled with a single quadrupole mass spectrometer ISQ QD^®^, both from Thermo Scientific, Waltham, MA, USA). The column for the chromatographic separation was a non-polar DB5 capillary column (0.25 mm i.d., 30 m length, supplied by Agilent, Santa Clara, CA, USA). The oxime derivative (*m*/*z* = 225) and 1,2 dibromopropane (*m*/*z* = 121), used as internal standards, were identified as target analytes and employed for the quantification of the formaldehyde concentration using a calibration curve derived from solutions at different but known formaldehyde concentrations. For each data point, an average value based on three replicates was employed to analyze the trends of the different tests.

### 2.3. Nanostructured Membranes Preparation

A 5%wt solution of PAN in DMF was prepared and then electrospun on a rotating drum collector. After that, a suitable amount of fibers was collected, the electrospinning was stopped, and electrospraying of the proper catalytic system suspension was performed. The optimized conditions for all the deposition processes are shown in Table 1, according to a previous study [45], and identified in order to avoid excessive catalyst agglomeration with membrane clogging and to obtain tailored amounts on the membrane surface.

The electrospray deposition of the catalytic systems was realized with 5% *w*/*w* concentrated suspensions in ethanol. Before deposition, the catalysts dispersions were preliminarily sonicated in an ice-cold bath for 40 min at a 40% amplitude using a 500 W ultrasonic probe (Vibra-cell VC505^®^ of Sonics & Materials, Newtown, CT, USA), alternating 1 min of sonication with 1 min of non-sonication to avoid solvent evaporation. Subsequently, Dynasylan^®^ 4144 was added at a 1% *w*/*w* concentration based on the ethanol content, followed by a further sonication of the resultant dispersion for 15 min. For the following analysis and discussion of the results, the membranes realized are named PAN_TiO_2_, PAN_Fe, and PAN_TiO_2__Fe, according to the fact that the deposited catalytic systems were TiO_2_, FeSO_4_, and TiO_2_ + FeSO_4_, respectively.

### 2.4. Set-Up for the Formaldehyde Degradation Tests

The set-up for the degradation tests is reported in detail in our previous work [45]. Briefly, about 70 mL of solution containing approximately 90 ppm of formaldehyde was placed in a Petri dish along with a 50 cm^2^ nanostructured membrane of PAN fibers covered by the electrosprayed particles of the specific catalytic system. The container with the formaldehyde solution was then suspended inside an ultrasonic bath (Branson Bransonic 1510, Emerson Electric, Ferguson, MI, USA) with a consumption power of 70 W and a working frequency of about 42 kHz. For the tests involving the use of UV radiation, a 16 W UVC lamp (UV Stylo E16, Light Progress, Anghiari, Italy), with $\lambda_{\max}$ = 254 nm, was placed on top of the ultrasonic bath and at a distance of 5 cm from the Petri dish, using a parallel configuration with respect to the surface of the membrane. During all catalytic experiments, the temperature was controlled by a continuous circulation of the water contained in the sonication bath.

## 3. Results and Discussion

### 3.1. Membrane Analysis

**Figure 1:**
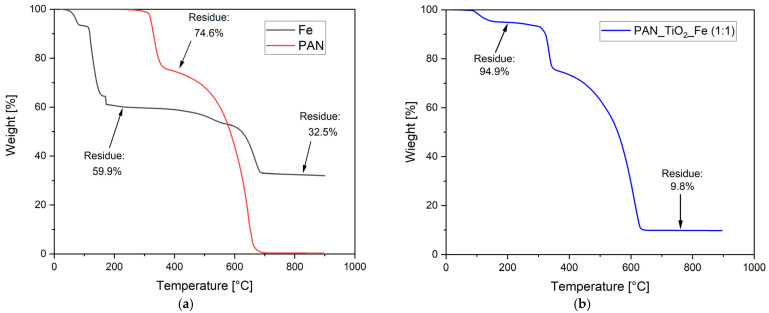
Weight residues of (**a**) PAN, FeSO_4_$\cdot {7H}_{2}O$ and (**b**) PAN_TiO_2__Fe (1:1) under air atmosphere.

Thermogravimetric analysis was carried out to quantify the amounts of the different catalytic systems deposited by electrospraying in order to compare the results of the abatement tests among the different membrane systems as a function of the catalyst weight. In order to assess the relative quantities of the catalysts employed in the case of the mixed composition (TiO_2_ + FeSO_4_), a first analysis was carried out in terms of the typical degradation profiles of pure materials. As can be seen in Figure 1a, PAN thermo-oxidative degradation, with a specific heating rate employed (20 °C/min) for the analysis, is a two-step process that can be identified by the mass-loss intervals of the polymer as a function of temperature [49]. The material mass was stable up to 250 °C, with an onset degradation temperature of about 300 °C, above which the decomposition of the material can be reasonably ascribed to a combination of reactions involving dehydrogenation, cyclization, and oxidation. The latter two are recognized to occur simultaneously, generating an unstable and partially hydrogenated cyclic structure, while oxidation, despite preceding cyclization, is characterized by a slower rate and becomes predominant in the second degradation stage, which involves PAN cyclization products [50,51]. Due to the high heating rate adopted in the test, the material was not stabilized, as in the case of carbon fiber pretreatment [52], and therefore, above 400 °C, the temperature was able to promote chain scission and the generation of volatile products up to the complete mass loss [53]. TiO_2_ is a stable material with a negligible mass loss (around 2%) at low temperatures due to the evaporation of adsorbed water, which can be considered not relevant for the membranes developed due to the relatively low amount of TiO_2_ deposited. On the other hand, the thermal degradation of FeSO_4_ is known to proceed with two different and subsequent degradation steps, divided into dehydration and decomposition [54,55]. In the first phase, crystallization water is lost upon heating at relatively low temperatures, producing anhydrous FeSO_4_ according to the following Equations (1)–(3) [56,57]:

(1) $${\mathrm{FeSO}}_{4}\cdot {7H}_{2}O\overset{40–90\;{}^{\circ}C}{\to }{\;\mathrm{FeSO}}_{4}\cdot {4H}_{2}{O+3H}_{2}O$$

(2) $${\mathrm{FeSO}}_{4}\cdot {4H}_{2}O\overset{140–200\;{}^{\circ}C}{\to }{\;\mathrm{FeSO}}_{4}\cdot H_{2}{O+3H}_{2}O$$

(3) $${\mathrm{FeSO}}_{4}\cdot H_{2}O\overset{270–350\;{}^{\circ}C}{\to }{\;\mathrm{FeSO}}_{4}{+H}_{2}O$$

Above 500 °C, FeSO_4_ starts to decompose by releasing SO_2_ up to 700 °C, and it is converted in Fe_2_O_3_ according to Equations (4) and (5) [58]:

(4) $${6\;\mathrm{FeSO}}_{4}\overset{525–650\;{}^{\circ}C}{\to }{\;\mathrm{Fe}}_{2}{\left({\mathrm{SO}}_{4}\right)}_{3}{+2\;\mathrm{Fe}}_{2}O_{3}{+3\;\mathrm{SO}}_{2}$$

(5) $${\mathrm{Fe}}_{2}{\left({\mathrm{SO}}_{4}\right)}_{3}\overset{625–710\;{}^{\circ}C}{\to }\;{\mathrm{Fe}}_{2}O_{3}+{3\;\mathrm{SO}}_{2}+\frac{3}{2}O_{2}$$

As can be seen in the thermogram of pure FeSO_4_ in Figure 1a, there were three distinct but partially overlapping mass losses up to 200 °C that can be associated with the dehydration reactions. Subsequently, the mass of the sample remained stable up to 400 °C, when the decomposition reaction occurred, according to the reactions suggested in the previous equations.

From the stoichiometry of the dehydration and decomposition reactions, it was possible to quantify the amount of volatile products released from the thermo-oxidative process and the theoretical residue, which was approximately 28.7%. Such a residue was slightly different from the final result, reported in Figure 1a, of about 32.5%. This could be explained as a partial loss of crystallization water during storage as the first dehydration step starts at a very low temperature. Indeed, according to the total mass loss at 800 °C, the true water content of the sample corresponds approximately to a FeSO_4_$\cdot {5H}_{2}O$ composition. This conclusion is in accordance with the fact that the first mass-loss step is around 6.8%, and it is close to the theoretical loss of 7.4% for the reaction ${\mathrm{FeSO}}_{4}\cdot {5H}_{2}O$ → FeSO_4_$\cdot {4H}_{2}O$ + H_2_O, while the total dehydration (40.1% mass loss) is in reasonable agreement with the theoretical value of 37%. Due to this evidence, the values obtained from the TGA were taken as a reference for the evaluation of the relative content of iron sulfate during electrospray deposition.

The introduction of FeSO_4_ in the membranes altered the degradation profile between the first and second mass-loss steps as Fenton reagents can act as hydroperoxide-decomposing agents during the formation of organic radicals [59,60,61], promoting a faster degradation of PAN and, therefore, a lower decomposition temperature. However, by comparing the TGA curves of the catalysts with that of the composite membrane PAN_TiO_2__Fe (Figure 1b), the initial weight loss of the latter up to 200 °C, where both TiO_2_ and PAN were quite stable (i.e., weight loss of less than 1%), can be related to the dehydration of FeSO_4_, while the final residue at 800 °C was due to residual Fe_2_O_3_ derived from FeSO_4_ oxidation and TiO_2_. On the basis of these considerations, it was possible to assess the relative content of the catalysts deposited on the membranes and the relative mass ratio between the inorganic components of the catalytic systems. Table 2 summarizes the results of these calculations in terms of the catalyst concentration and composition ratios of the different membranes employed in the tests.

After tailoring the deposition amount for the different catalytic systems, it was possible to control the overall amount deposited on the membranes and the relative composition between the FeSO_4_ and TiO_2_ whenever they were deposited on the same membrane. Since the TGA results in Table 2 show that the overall quantities were quite similar to each other, it was decided to test these systems to compare the abatement of formaldehyde without the necessity of rescaling the amount of pollutant degraded on the catalyst content present on the surface of the membranes.

### 3.2. Photocatalytic Tests

Following the results of our previous work [45] on the combined use of different AOPs, we tried to improve the HCHO abatement performance of composite nanofibrous membranes by introducing the effect of ultrasound. Such a solution was selected to obtain improvements in terms of the mass transfer on the surface of the catalytic systems employed and to evaluate if ultrasound could also provide a chemical effect to promote the degradation of HCHO. To such an end, the first analysis involved the effect of the neat ultrasound action on HCHO in the absence of any catalyst and membrane support, in comparison to the effect of UV radiation and the combination of these two processes.

**Figure 2:**
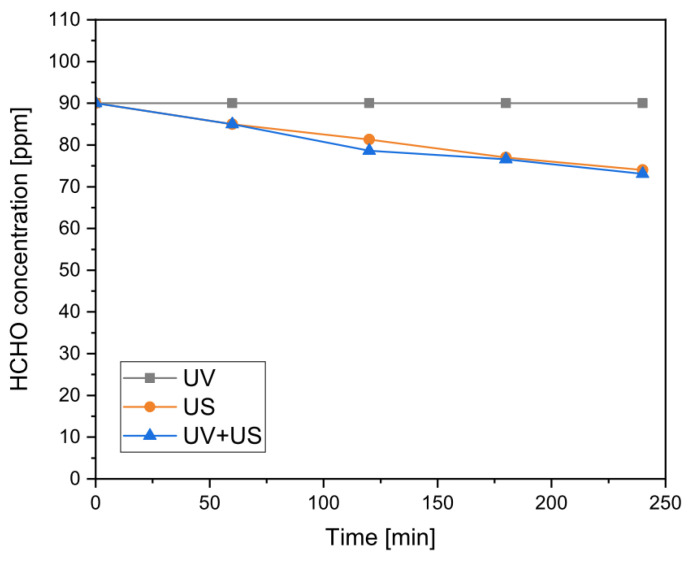
Residual concentration of formaldehyde (HCHO) over time for ultra-violet (UV), ultrasound (US), and UV+US tests (maximum standard deviation, σ_max_ = 1.3 ppm).

The results of these tests are shown in Figure 2, where it is possible to observe that the UV radiation alone did not provide any effect on the residual concentration of HCHO, and therefore, in the absence of any photocatalyst, it was not able to induce photodecomposition of the pollutant. On the other hand, ultrasound showed the ability to induce a limited but constant degradation of HCHO over time, reaching a final conversion of about 20% over the course of 4 h of treatment, quite independently from the presence of UV light. On the basis of these results, it is possible to confirm that there was no relevant synergic effect due to the coupling of the two techniques and that the profile of the concentration of HCHO was only affected by the action of ultrasound. Such an effect, at a relatively low US frequency, was also noted by Navarro et al. [62] for formic acid and was explained as the direct consequence of water sonolysis (Equations (6)–(11), [63]) and the radical scavenging action of the pollutant on the produced ^•^OH species.

H_2_O + ))) → H^•^ + ^•^OH (6)

^•^OH + ^•^OH → H_2_O_2_ (7)

^•^OH + ^•^OH → H_2_ + O_2_ (8)

H^•^ + O_2_ → HO_2_^•^ (9)

H^•^ + HO_2_^•^ → H_2_O_2_ (10)

HO_2_^•^ + HO_2_^•^ → H_2_O_2_ + O_2_ (11)

A further “blank” test was realized with a PAN-TiO_2_ membrane in the absence of US and UV action to probe any effect related to the photocatalyst, but only a slow decrease in the concentration was detected over the course of the test and attributed to surface adsorption of the pollutant.

**Figure 3:**
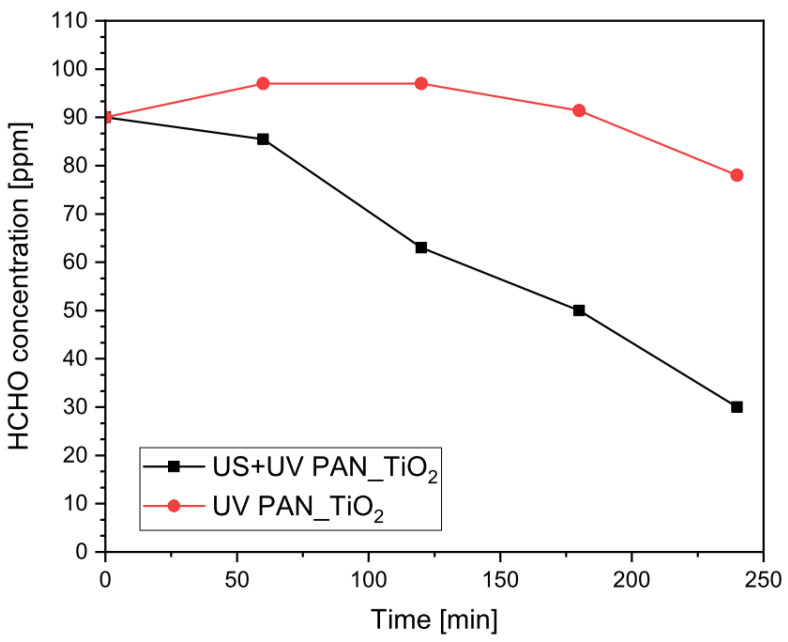
Residual concentration of HCHO for photocatalysis and sono–photocatalysis tests (σ_max_ = 1.9 ppm).

However, as can be noted in Figure 3, when photocatalysis was coupled with ultrasound (US + UV PAN_TiO_2_), the degradation of the pollutant was more efficient since it did not increase the initial concentration of formaldehyde, as was the case with an absence of ultrasound (UV PAN_TiO_2_).

This initial increase in the formaldehyde content during a photocatalytic test, without US, was explained as a consequence of the partial oxidation of methanol, used as a commercial stabilizer from the starting formalin solution, that generates formaldehyde as an intermediate degradation product due to the reaction with dissolved oxygen (Equations (12)–(13), [8]):

CH_3_OH + ^•^OH → ^•^CH_2_OH + H_2_O (12)

^•^CH_2_OH + O_2_ → CH_2_O + HO_2_^•^ (13)

Due to the non-negligible amount of methanol in the formalin solution (about 25 ppm per 90 ppm of formaldehyde), a specific photocatalytic experiment was also carried out to test this hypothesis, using an aqueous solution containing 25 ppm of methanol. The results (not shown) confirm the proposed mechanism with a progressive increase in formaldehyde formation up to a maximum value after 120 min, in accordance with the findings of Araña et al. [8]. This allows for the hypothesis that methanol is preferentially adsorbed on the surface of hydrophilic nanoparticles of P90 and more rapidly photo-oxidized than formaldehyde, increasing its concentration over time. From this point of view, the change in the formaldehyde concentration profile over the course of the reaction promoted by the presence of ultrasounds can be a consequence of a different degradation mechanism. Indeed, as observed by Rasshokin et al. [64], in the sonolysis of diluted aqueous methanol solutions, methanol can be converted into methane during ultrasound treatment, according to the following Equations (14)–(17) in which thermolysis of the C-O bonds is coupled with the radical species produced from water sonolysis [63]:

CH_3_OH → ^•^CH_3_ + ^•^OH (14)

^•^CH_3_ + H^•^ → CH_4_ (15)

^•^CH_3_ + HO_2_^•^ → CH_4_ + O_2_ (16)

^•^CH_3_ + H_2_ → CH_4_ + H^•^ (17)

It has to be underlined, however, that those conclusions cannot be strictly applied to the present case as the sonication frequency was significantly different (724 kHz versus 42 kHz) and also because other elementary reactions, which can lead to the formation of formaldehyde, had to be taken into consideration (Equations (18)–(21), [65]):

CH_3_OH → ^•^CH_2_OH + H^•^ (18)

CH_3_OH + H^•^ → ^•^CH_2_OH + H_2_ (19)

^•^CH_2_OH + H^•^ → CH_2_O + H_2_ (20)

^•^CH_2_OH + ^•^OH → CH_2_O + H_2_O (21)

In this sense, it was explained [64] that in diluted solutions of methanol, the possible formation of formaldehyde is only due to the migration of H^•^ and ^•^OH radicals to the bulk of the aqueous solution. If the formaldehyde concentration during the experiments does not increase, as when US and photocatalysis are coupled, it is likely that the extent of migration within the bulk of the liquid phase is limited. As a consequence, the H^•^ and ^•^OH radicals recombine or react with methanol at the bubble interface where the temperature reaction is higher and the ^•^CH_2_OH radical produced from methanol can be converted into CO (Equations (22)–(24), [66]):

^•^CH_2_OH + M → CH_2_O + H^•^ + M (22)

CH_2_O + M → H^•^ + ^•^CHO + M (23)

^•^CHO + M → CO + H^•^ + M (24)

It can therefore be concluded that the high temperatures reached in the cavitation bubbles during the exposition to ultrasound might have provided a different set of reactions involved in methanol degradation, at least in the absence of a concurrent effect of a photocatalytic nature. In the latter case, a further explanation of the result could be related to the TiO_2_-promoted production of different active species (i.e., HO_2_^•^ and ^•^OH) due to the photocatalytic decomposition of H_2_O_2_ [67] derived from water sonolysis (Equations (6)–(11)), possible even at low ultrasonic frequencies [68]. A higher concentration of such active species can promote the reaction with both methanol and formaldehyde, possibly explaining the lower initial degradation rate observed within the first 60 min of the reaction as a consequence of the balance between formaldehyde degradation and production from methanol oxidation. The relevance of the two proposed mechanisms could be verified with proper analytic techniques, such as electron spin resonance [69,70]; however, this was outside of the main aim of this work. It is, however, clear that such improved reactivity, coupled with the higher mass transfer improvement observed with the use of ultrasound in heterogeneous reacting systems [71,72], can enhance the overall efficiency of the degradation process and possibly explain the constant decrease in the pollutant concentration.

**Figure 4:**
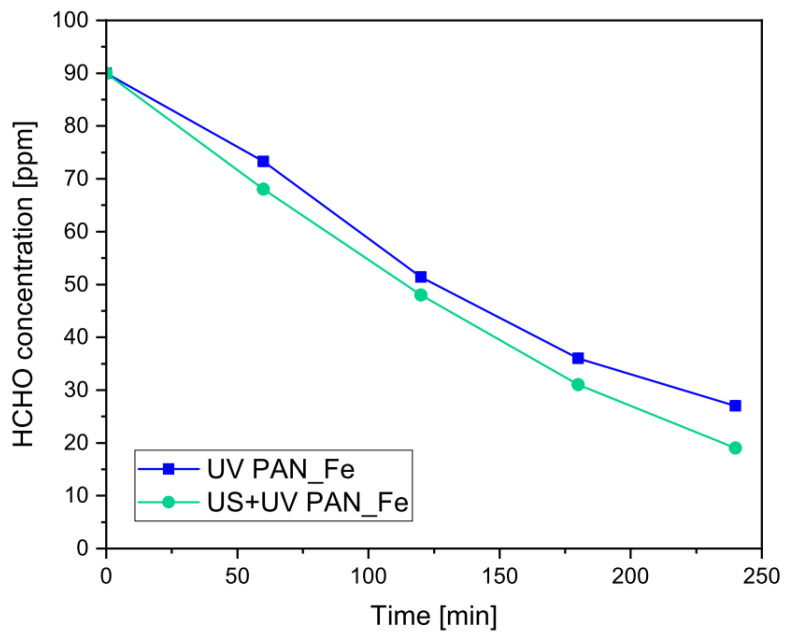
Residual concentration of HCHO over time for photo–Fenton and sono–photo–Fenton tests (σ_max_ = 1.3 ppm).

A second set of tests was carried out by combining the ultrasound irradiation with the photo–Fenton process, as shown in Figure 4. It is well documented that UV-C radiation is able to assist the regeneration of the Fenton cycle by the photo-reduction of ferric ions under acidic pH, increasing the efficiency of the process and reducing the consumption of H_2_O_2_ needed to sustain the process [73,74,75,76]. On the other hand, the aforementioned effect of the H_2_O_2_ concentration increase as a consequence of the introduction of ultrasound might directly promote the Fenton reaction, inducing a higher amount of radical species [77]. For the purpose of the present study, it was decided to take advantage of such an effect as a way to improve the efficiency of the formaldehyde degradation obtained in the absence of any added H_2_O_2_, as highlighted in our previous study [45].

**Figure 5:**
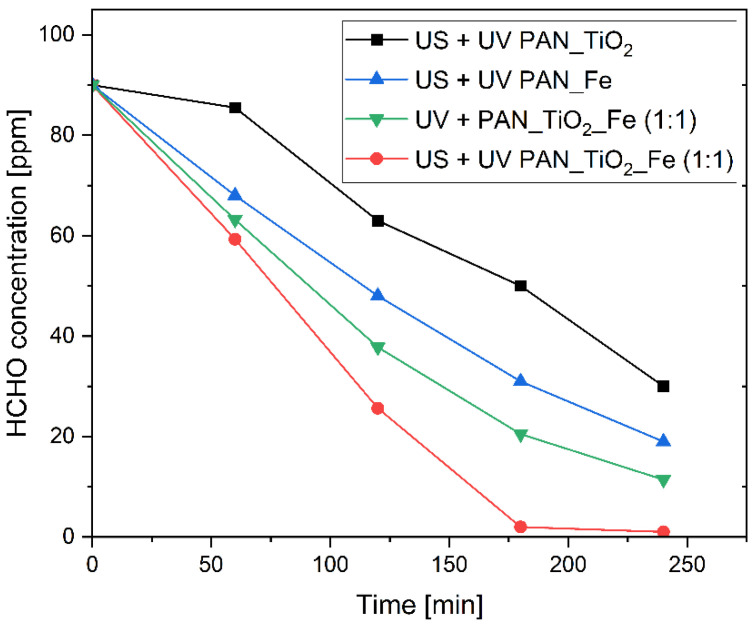
Comparison of the residual concentration trend of HCHO over time for tested advanced oxidation processes (AOPs) (σ_max_ = 1.9 ppm).

In this case, the results of the tests in Figure 4 show that the introduction of ultrasound can improve, although to a limited extent, both the rate and the final conversion in comparison to the photo–Fenton process, with an evident additive effect. This can be ascribed to the high initial degradation rate of the pure photo–Fenton reaction, which does not suffer from the limitations shown by the heterogeneous nature of the photocatalysis process. On the basis of these results, it is possible to affirm that the ultrasound had a beneficial effect for both processes, i.e., photocatalysis and photo–Fenton, although to different extents, and therefore, it was plausible to analyze the performance of abatement due to the combination of these different AOPs in a single system, realizing the sono–photocatalytic–Fenton abatement of formaldehyde. The results of these tests are shown in Figure 5, in comparison with other tested systems, where the sono–photocatalytic–Fenton system was employed using a tailored deposition of FeSO_4_ and TiO_2_ in order to respect a Fe/TiO_2_ mass composition ratio of 1:1. This may provide a simpler explanation if the combined process shows a synergic or simply additive effect; as already reported before, the total amount of catalysts was about the same in all the tested systems.

As can be seen, there were distinct performances of the different systems. In particular, the trends with the best results were ranked as follows: sono–photocatalytic–Fenton > photocatalytic–Fenton > sono–photo–Fenton > sono–photocatalysis. This ranking reflects the improvements both in the initial degradation rate, calculated as the average value from the data in the 0–120 min range, and the final conversion (Table 3), indicating that the contribution of the ultrasound improved each single AOP.

A possible explanation of the contribution provided by ultrasounds can be understood not only in terms of higher mass transfer conditions but also as a way for promoting further pollutant degradation by both ^•^OH and H_2_O_2_ formation [63].

On the one hand, such H_2_O_2_ can be photo-catalytically decomposed by UV radiation to generate a new active radical species that can oxidize the organic pollutant, and on the other hand, it can be involved in a synergic cascade effect. Indeed, while iron is able to limit the electron–hole recombination of TiO_2_, the H_2_O_2_ derived from water sonolysis can be involved in promoting the iron oxidation from Fe(II) to Fe(III) [78,79], while UV radiation allows for the photo-reduction of Fe(III) to Fe(II) in order to restart the Fenton cycle [80]. This effect could be important for producing H_2_O_2_ in situ without any addition of the oxidant at the beginning of the process, although the relevance of such an effect has to be carefully evaluated as a function of the pollutant concentration and processing conditions.

A further consideration can be made in terms of the possible products derived from formaldehyde oxidation according to the different findings reported in the literature [40,41,45,70,81]. For the present case, due to the nature of the processes involved and the liquid phase medium, the probable reaction intermediate was formic acid, which can be subsequently oxidized to CO_2_. Such an intermediate has commonly been reported for the photocatalysis, Fenton, and photo–Fenton processes in aqueous solutions [40,41] and is consistent with the pH fluctuation observed over the course of the reaction [45]. On the other hand, the effect of ultrasound in combination with photocatalysis on product distribution, although not specifically addressed in the literature, has been proven not to inhibit the degradation of HCHO for low concentrations in aqueous solutions [38] and, therefore, does not represent a main concern for the present study.

**Figure 6:**
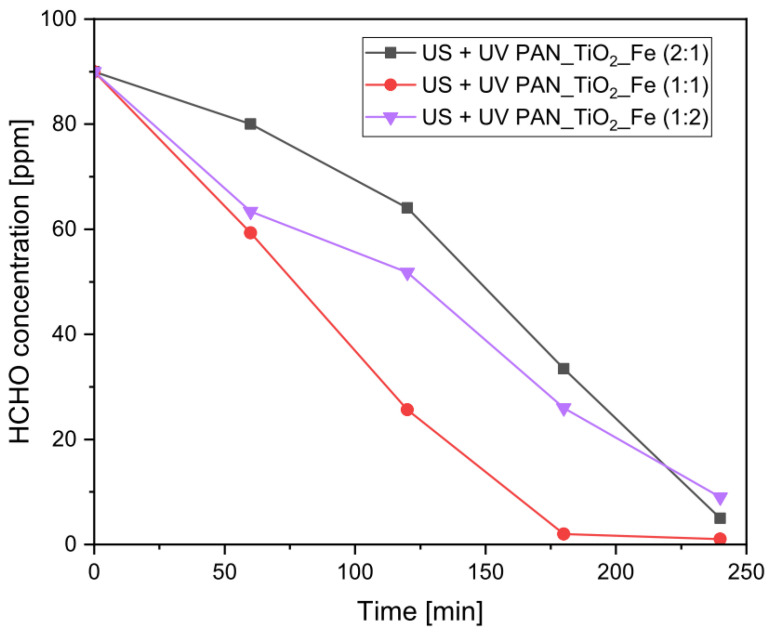
Comparison of the residual concentration trend of HCHO for the sono–photocatalytic–Fenton systems with different TiO_2_/FeSO_4_ mass ratios (σ_max_ = 2.3 ppm).

Due to the good results obtained with the employment of the sono–photocatalytic–Fenton process, a further analysis regarding the effect of the composition ratio between the TiO_2_ and FeSO_4_ was carried out. According to the previous synergic effect, altering the composition ratio may have led to a different contribution to the catalytic activity of the systems. In this sense, Figure 6 reports the abatement profiles of the sono–photocatalytic–Fenton systems for different Fe/TiO_2_ mass ratios in comparison to the 1:1 reference composition.

The results show that the optimal performance, in terms of the abatement rate, occurred when the relative composition ratio was 1:1. The second-highest performing system was the one with a higher FeSO_4_ content (1:2), which, however, over the course of the experimental time analyzed, provided a similar final conversion as the system with a higher TiO_2_ content (2:1). It is evident that altering the composition of the catalytic system influenced the HCHO abatement performance as a consequence of the different kinetics of the involved processes. The lower abatement rate, with a 1:2 mass ratio composition, can be explained by the higher FeSO_4_ relative content shielding the UV absorption of TiO_2_ and reducing its photoactivity, similar to the trend of the sono–photo–Fenton process, but with a more limited final conversion due to the lower iron content. On the other hand, increasing the amount of TiO_2_ (a 2:1 mass ratio) worsened the abatement rate because the photocatalytic process, characterized by lower kinetics also in the presence of US, became predominant over the photo–Fenton reaction and slowed down the overall rate of the process.

## 4. Conclusions

The present work investigated the effects of the combinations of different AOPs on the degradation of aqueous solutions with a low concentration (90 ppm) of formaldehyde. Several strategies based on the employment of UV photocatalysis, the Fenton reaction without added H_2_O_2_, and ultrasound irradiation were considered with the use of heterogeneous membranes made of electrosprayed TiO_2_ and FeSO_4_ particles supported on electropsun PAN nanofibers. The results show that ultrasound can improve the kinetics and final conversion of formaldehyde, overcoming the limitations of photocatalysis, with a synergic effect possibly related to the higher mass transfer efficiency and combined degradation promoted by sonolysis. This effect was less marked in the case of the sono–photo–Fenton reactions since the photo–Fenton process is characterized by faster kinetics, and the contribution to the trend of conversion over time was rather additive than synergic. The combination of the different AOPs for the sono–photocatalytic–Fenton abatement tests showed that it was possible to combine the previous improvements to obtain a system that was able to provide a 99% decrease in the formaldehyde concentration in 240 min. However, this effect was achieved only when the relative concentrations of the Fe and TiO_2_ were balanced to allow for a synergic contribution and avoid a possible photo-shielding effect from the Fe source or predominance of the photocatalysis over the better-performing photo–Fenton reaction. Such findings can provide new detailed insight into the rationale of combining efficiently different AOPs and overcoming the limitations that affect each one of these processes.

## Figures and Tables

**Table 1:** Deposition parameters for membrane production.

System	Flow Rate (mL/h)	Voltage (kV)	Electrodes Distance (cm)	Relative Humidity (%)	Deposition Time (min)
PAN support	2	23	20–25	30–40	120
Catalyst deposition	2	23	15	20–30	variable

**Table 2:** Residues, relative compositions, and catalyst concentrations derived from the thermogravimetric (TGA) analysis of the different catalysts and membranes employed for the abatement test.

Sample	Residue @200 °C (%)	Residue @800 °C (%)	FeSO_4_ Content (%)	TiO_2_ Content (%)	Fe:TiO_2_ Ratio	Catalyst Conc. (mg/cm^2^)
TiO_2_	98.8	98.0	-	100	-	-
Fe	59.9	32.5	100	-	-	-
PAN_TiO_2_	99.8	17.6	-	17.6	-	0.141
PAN_Fe	94.4	4.7	14.5	-	-	0.124
PAN_TiO_2__Fe (2:1)	94.9	16.1	7.7	13.6	0.56	0.135
PAN_TiO_2__Fe (1:1)	94.9	9.8	7.7	7.3	0.94	0.133
PAN_TiO_2__Fe (1:2)	94.6	7.1	8.1	4.5	1.81	0.129

**Table 3:** Initial degradation rate and final conversion of HCHO for the different combined AOPs tested.

System	Process	Initial Degradation Rate (ppm/min)	Final Conversion (%)
US + UV PAN_TiO_2_	Sono–photocatalysis	0.195	67
US + UV PAN_Fe	Sono–photo–Fenton	0.353	79
UV + PAN_TiO_2__Fe	Photocatalytic–Fenton	0.437	87
US + UV PAN_TiO_2__Fe (1:1)	Sono–photocatalytic–Fenton	0.531	99

## Data Availability

Data are contained within the study.
